# Medical students’ awareness of health issues, attitudes, and confidence about caring for lesbian, gay, bisexual and transgender patients: a cross-sectional survey

**DOI:** 10.1186/s12909-020-02409-6

**Published:** 2021-01-14

**Authors:** Sophie Arthur, Abigail Jamieson, Harry Cross, Kate Nambiar, Carrie D. Llewellyn

**Affiliations:** 1 Department of Primary Care and Public Health, Brighton and Sussex Medical School, Watson Building, Village Way, Falmer Campus, Brighton, BN1 9PH UK; 2 Brighton and Sussex University Hospitals NHS Trust, Eastern Road, Brighton, BN2 5BE UK

**Keywords:** Medical students, LGBT, Education, Curriculum, Attitudes, Patients

## Abstract

**Background:**

Lesbian, gay, bisexual and transgender (LGBT) patients have an increased incidence of a range of health problems, and face many barriers to accessing healthcare. Our research aimed to explore the awareness of health issues and attitudes of medical students towards LGBT patients’ health including barriers to health services, their attitudes towards inclusion of LGBT content in the curriculum and their confidence with providing care for their LGBT patients in the future.

**Methods:**

Medical students were recruited to take part in a cross-sectional survey. We used a 28-item survey to explore views about the undergraduate medical curriculum.

**Results:**

252 surveys were analysed from 776 eligible participants. Attitudes towards LGBT patients were positive but awareness and confidence with respect to LGBT patients were variable. Confidence discussing sexual orientation with a patient significantly increased with year of study but confidence discussing patient gender identity did not. The majority of participants (*n* = 160; 69%) had not received specific training on LGBT health needs, and 85% (*n* = 197) wanted to receive more training.

**Conclusions:**

Increasing the amount of LGBT teaching in undergraduate medical curricula could help to increase the quality of doctor-patient interactions, to facilitate patients’ disclosure of sexual orientation and gender identity in healthcare and increase the quality of healthcare.

## Background

In the UK, an estimated 2.5% of people identify as lesbian, gay or bisexual (LGB) [1] although this is likely to be an underestimate. Approximately 1% of people identify as transgender, with increasing numbers of referrals to gender identity clinics [2, 3]. The LGBT community is a heterogenous group, with varied demographics, sexual orientations, gender identities and behaviours. Therefore, subgroups within the LGBT community are unlikely to share the same health needs [4]. Nevertheless LGBT (plus queer (Q) and intersex (I)) groups have been historically considered as representing a wide range of individuals who are likely to have experienced marginalisation and stigma. Particular subgroups may have higher risks of a variety of physical, sexual and mental health issues. Physical health problems include increased risks of cardiovascular disease, obesity, and certain cancers such as testicular, prostate, breast and endometrial. Sexual health problems include increased rates of sexually transmitted infections including HIV and experience of sexual violence [5, 6]. Depression, self-harm anxiety and suicide are more prevalent within LGBT groups [5, 7, 8]. The UK National LGBT survey reported that 24% of participants had accessed mental health services in the previous 12 months [9]. Furthermore, LGBT people have higher incidences of substance use, family rejection, homelessness and isolation; all social determinants of health [10]. Minority stress theory hypothesises that long term discrimination and stigma can lead to chronic stress, potentially resulting in long-term physical and mental health problems [11].

The increased incidence of these conditions in the LGBT population reinforces the need for good access to healthcare services. However, LGBT patients face many barriers to healthcare. Systemic barriers include insensitive or lack of screening invitations, a lack of specialised services such as gender identity clinics, or deliberate withholding of treatment [3, 7, 10, 12, 13]. Communication barriers can arise in situations where a clinician lacks awareness or has negative views towards LGBT patients. This can result in discrimination, presumptive questions or insensitive remarks relating to sexual orientation or gender identity, for example, moral judgements and inappropriate use of gender pronouns [7, 12, 14]. Five percent of British LGBT patients had been encouraged to access services aimed at challenging their sexual orientation or gender identity [15]. A national UK survey of transgender patients who had accessed healthcare services in the last 12 months found that 38% reported a negative consultation because of their gender identity, 21% did not have their specific health needs acknowledged, and 18% feared accessing health services because they felt they would be discriminated against [7].

Educating healthcare professionals (HCPs) about LGBT-related healthcare issues is considered the most effective way to improve LGBT patients’ engagement in healthcare [16]. Mental health staff who had LGBT awareness training were more likely to discuss problems relating to the patients’ sexual orientation or gender identity in their consultations [16]. Being part of the LGBT communities can be a determinant of health, and understanding the wider social determinants of health is outlined in the General Medical Council’s (GMC) outcomes for graduates [17]. Despite this, LGBT-specific teaching across medical schools is limited. A US survey completed by 132 medical schools found that the median time dedicated to specific LGBT teaching was five hours in the entire curriculum [18]. Recent research from the UK has shown that of students from one UK medical school, only 15% agreed that they had received any LGBT specific training [12]. This study failed to assess the unique issues of sexual orientation or gender identity separately and the research on LGBT teaching in UK medical schools is generally sparse. Furthermore, a systematic literature review concluded that addressing these issues as part of undergraduate medical curricula is an essential step to breaking healthcare related barriers [14] and has a positive effect on HCPs knowledge and attitudes [19].

We had two objectives for the study. The first was to assess the awareness of health issues and attitudes of medical students’ towards LGBT healthcare issues as well as their self-reported confidence in treating LGBT patients. Our second objective was to assess the attitudes of the medical students towards the amount and content of teaching they currently receive on LGBT health issues.

## Methods

### Study design

Cross-sectional survey.

### Participants

Undergraduate students enrolled on a Bachelor of Medicine, Bachelor of Surgery (BMBS) degree at one medical school.

### Recruitment

All 776 medical students (years one to five) registered at one medical school in the South East of England were eligible, including those enrolled on an intercalated degree. The following numbers in each year were eligible: year 1–162; year 2–134; year 3–142 (including an additional 54 intercalators; year 4–126 and year 5–158. The recruitment period was September 2018 to January 2019.

### The survey

A 28-question cross-sectional survey was created. This consisted of six demographic questions, eighteen specially devised questions and four questions adapted from previously validated surveys [12, 20]. Section one was a short series of demographic-related questions (Table 1). Section two (Part A) assessed self-reported confidence in treating LGBT patients using a battery of items from Parameshwaran et al. [12] For example, *“How confident do you feel taking a sexual history from an LGB&T patient?”*, scored on a Likert scale from ‘very unconfident’ to ‘very confident’ (1–5). Three items assessed students’ skills and confidence in general, for example, *“how confident do you feel taking a sexual history from a patient?”,* in order to understand whether any lack of confidence was due to the skill itself or the performance of the skill with an LGBT patient. Two additional items (Part B) were included in section two: participants ranked their comfort level regarding asking a patient about their sexual orientation or gender identity. These questions were scored (1–5) from ‘strongly disagree’ to ‘strongly agree’, in relation to the statements with the stem “*I would feel comfortable* …” (Table 2).

**Table 1:** Demographic characteristics of participants (*n* = 252)

**Training**		**Sexuality**	
Year 1	57 (22.6%)	Heterosexual	202 (80.2%)
Year 2	44 (17.5%)	Gay or lesbian	14 (5.6%)
Year 3	51 (20.2%)	Bisexual	29 (11.5%)
Intercalating	20 (7.9%)	Asexual	1 (0.4%)
Year 4	44 (17.5%)	Other orientation not listed	6 (2.4%)
Year 5	36 (14.3%)	**Religion**	
**Gender**		No religion	142 (56.3%)
Male	94 (37.3%)	Buddhist	8 (3.2%)
Female	158 (62.7%)	Christian	63 (25.0%)
Non-binary	0 (0.0%)	Hindu	11 (4.4%)
Other	0 (0.0%)	Jewish	3 (1.2%)
**Gender same as assigned at birth**		Muslim	19 (7.5%)
Yes	250 (99.2%)	Sikh	4 (1.6%)
No	2 (0.8%)	Any other religion	2 (0.8%)
		Median age (Q_1−_Q_3_)	22 (20–23)

**Table 2:** Self-reported confidence (*n =* 252)

Part A ‘Please rate from very unconfident to very confident’	Median (Q_1−_Q_3_)	
How confident do you feel clarifying unfamiliar sexual or gender terms used by patients?^a^	3 (2–4)	
How confident do you feel taking a social history from a patient?	4 (4–5)	
How confident do you feel taking a social history from an LGB&T patient?^a^	4 (3–4)	
How confident do you feel taking a sexual history from a patient?	4 (3–4)	
How confident do you feel taking a sexual history from an LGB&T patient?^a^	4 (3–4)	
In a situation where domestic violence would be considered a possibility, how confident do you feel discussing this with a patient?	2 (2–3)	
In a situation where domestic violence would be considered a possibility, how confident do you feel discussing this with an LGB&T patient?^a^	2 (2–3)	
How confident do you feel deciding which ward (e.g. male/female ward) a transgender patient should be nursed?^a^	2 (2–4)	
How confident do you feel knowing where to look in order to find information about LGB&T-specific health services in your area?^a^	2 (2–3)	
**Part B** Statement	Median (Q_1−_Q_3_)	Kruskal-Wallis H
*“I would be comfortable at my stage of medical school education asking a patient about their sexual orientation if I thought it was relevant or necessary”*	Year 1 4 (3–4) Year 2 4 (3.5–4) Year 3 4 (4–4) Year 4 4 (4–5) Year 5 4.5 (4–5)	(H (4)=26.999, *p =* 0.001)
*“I would be comfortable at my stage of medical school education asking a patient about their gender identity if I thought it was relevant or necessary”*	Year 1 4 (3–4) Year 2 4 (3–4) Year 3 4 (3–4) Year 4 4 (3–5) Year 5 4 (4–4)	(H (4)=5.989, *p =* 0.2*) (NS)*

**Part A:** Questions scored 1–5; 1 (Very unconfident), 2 (Unconfident) 3 (Neither unconfident nor confident), 4 (Confident), 5 (Very confident). ^a^Taken from Parameshwaran et al. [12]

**Part B:** Questions are scored from 1 to 5; 1 (Strongly disagree), 2 (Disagree), 3 (Neutral), 4 (Agree), 5 (Strongly agree). *NS- Not significant*

Section three assessed attitudes toward LGBT patients using 10 items [12]. Participants stated their level of agreement with each statement on a Likert scale scored 1–5 from “strongly disagree” to “strongly agree”. For example, “*Same-sex behaviour is a natural expression of sexuality in humans”*. This was modified from Parameshwaran et al by adding five items and editing three others to clearly separate LGB and transgender statements (Table 3) [12]. Following this a series of items asked how common participants thought various health issues were for LGB or transgender patients compared to heterosexual or cisgender patients. These items were scored 1–9, with higher scores indicating health issues being “more common” and low scores being “less common” for LGB or transgender patients (Table 4). The final item of section three asked participants to select any of the six provided reasons that might negatively affect an LGB or transgender patients’ attendance at healthcare services.

**Table 3:** Attitudes toward LGBT patients (*n* = 235)

“Please rate the following statements, from strongly disagree to strongly agree”	
Statement:	Median (Q_1−_Q_3_)
LGB&T patients deserve the same level of quality care from health services as heterosexual patients^a^	5 (5–5)
LGB&T patients should only seek health care from LGB&T health clinics^a^	1 (1–2)
LGB&T patients should disclose their sexual orientation to their physicians^a^	3 (2–4)
LGB&T patients should disclose their gender-identity to their physicians	3 (3–4)
I would be comfortable if I became known among my professional peers as a doctor who cares for LGB&T patients^a^	5 (4–5)
I would be comfortable telling my friends and family that I cared for LGB&T patients^a^	5 (4–5)
Same-sex sexual attraction is a natural expression of sexuality in humans^a^	5 (4–5)
A broad diversity of many different gender expressions and identities is natural in humans	5 (4–5)
It is more challenging to conduct a physical examination with an LGB patient than with a heterosexual patient^a^	1 (1–2)
It is more challenging to conduct a physical examination with a patient who identifies as transgender than with a cisgender patient	3 (2–4)
It is more challenging to conduct a genitourinary examination with an LGB patient than with a heterosexual patient^a^	1 (1–2)
It is more challenging to conduct a genitourinary examination with a patient who identifies as transgender than with a cisgender patient	3 (2–4)
It is more challenging to discuss sexual behaviour with LGB patients than with heterosexual patients^a^	2 (1–2)
It is more challenging to discuss sexual behaviour with transgender patients than with a patient who identifies as cisgender	2 (1–3)

Questions are scored from (1–5). 1 (Strongly disagree), 2 (Disagree), 3 (Neutral), 4 (Agree), 5 (Strongly agree) ^a^Questions adapted from Parameshwaran et al *(*Originally from Sanchez et al. [12, 20]

**Table 4:** Awareness of health issues amongst LGBT patients (*n =* 235)

“How common do you think the following health issues are for …”	LGB people compared to heterosexual people? Median (Q_1−_Q_3_)	Transgender people compared to cisgender people? Median (Q_1−_Q_3_)
Problematic alcohol use	6 (5–7)	7 (6–8)
Anxiety	7 (7–8)	8 (7–9)
Avoidance of health services	7 (6–8)	8 (7–9)
Not attending Breast Cancer screening	5 (5–7)	7 (6–8)
Not attending Cervical screening	5 (5–7)	7 (6–8)
Childhood diseases	5 (5–5)	5 (5–5)
Depression	7 (7–8)	8 (7–9)
Diabetes	5 (5–5)	5 (5–5)
Dissatisfaction with health services	7 (6–8)	8 (7–9)
Drug use	7 (5–8)	7 (6–8)
Eating disorders	6 (5–7)	7 (6–8)
Feeling welcome/ accepted in health services	3 (2–5)	3 (2–6)
Maternity/childbirth	5 (3–5)	4 (3–5)
Self-harm	7 (6–8)	7 (7–8)
Smoking	5 (5–7)	6 (5–7)
Suicidal thoughts and/or behaviours	7 (6–8)	8 (7–9)

Questions are scored from (1–9), 1 (Less common for people who are LGB/transgender), 5 (The same for people who are LGB/transgender), 9 (More common for people who are LGB/transgender)

Section four assessed familiarity with LGBT-related terminology using a table of 30 words [12]. We added three more recently used terms; ‘Chemsex’, ‘Crystal/Tina’ and ‘G/GHB’. Participants rated their understanding of these terms using a Likert scale scored 1–5. Higher scores indicated greater familiarity with the terms (Table 5).

**Table 5:** Proportion of students “confident” or “very confident” in their knowledge of the following terms (*n* = 232)

Term	% (n)	Term	% (n)
Asexual	**85.3%** (198)	Phalloplasty	**41.8%** (97)
Bisexual	**97.0%** (225)	Neovagina	**37.1%** (86)
Gay	**97.8%** (227)	“T” [Testosterone]	**72.8%** (169)
Genderqueer	**31.0%** (72)	Top surgery	**45.3%** (105)
Intersex	**37.5%** (87)	Bareback	**55.6%** (129)
Lesbian	**97.4%** (226)	BDSM	**75.4%** (175)
Pansexual	**56.9%** (132)	Douching	**55.2%** (128)
Polyamorous	**71.1%** (165)	Fisting	**81.9%** (190)
Queer	**41.4%** (96)	Rimming	**72.4%** (168)
Trans	**89.2%** (207)	Serosorting	**6.9%** (16)
Trans woman	**84.5%** (196)	Saunas	**37.5%** (87)
Trans man	**84.9%** (197)	Slamming	**25.0%** (58)
Transgender	**91.4%** (212)	Topping	**34.1%** (79)
Transitioning	**89.2%** (207)	Chemsex^a^	**60.8%** (141)
Bottom surgery	**37.1%** (86)	Crystal/Tina^a^	**26.7%** (62)
PEP^b^	**80.2%** (186)	G/GHB^a^	**42.2%** (98)
Dental dams	**37.1%** (86)		

^a^added by our research term, the remaining terms are from Parameshwaran et al. [12]

^b^Post-exposure prophylaxis

Section five enquired about the inclusion of LGBT health needs in the undergraduate curriculum. One item was adapted [12] asking participants to rank their level of agreement with the statement “*I have received specific training on LGB&T health issues in undergraduate teaching at medical school”*. Additional questions asked participants’ opinions of the amount of teaching they receive on LGB and transgender patients’ health needs, ranked from “too much” to “not enough” on 5-point scales. Participants were asked if they would be interested in receiving more teaching on LGBT-specific health issues, with yes or no tick boxes. Participants were then asked whether they would prefer this teaching in standalone modules or worked into the spiral curriculum. Finally, participants ranked five factors which had contributed to their knowledge of LGBT health problems from most to least, and ranked six options from ‘most beneficial’ to ‘least beneficial’ with respect to the type of teaching they would most like to see increased in the curriculum.

### Procedure

The online survey was hosted on Qualtrics and was sent to all eligible medical students as a link on an advertising email. A paper version was distributed by hand between student lectures. Prior to starting the survey, participants read a short information paragraph about the study and ticked a consent box for their anonymised data to be used. Data remained anonymous throughout collection. Participants could opt into a prize draw for £40 Amazon vouchers by providing an email address. The paper survey had a separate page for entry into a prize draw and this was detached from answers before collection. All online surveys were transferred to Statistics Package for the Social Sciences (SPSS) version 25 [21], without email addresses. Paper survey data was manually added to the same spreadsheet. The prize draw winner was selected using an online random number generator.

### Data analysis

All surveys, excluding surveys where only demographic questions were answered, were used in analysis. Missing data was deemed to be missing at random and were kept in the dataset allowing partial deletion by the software programme SPSS. Data from participants undertaking intercalated degrees in year 4 were merged with year 3 participants, as they had formally completed a similar amount of the curriculum. Where the data was non-normally distributed, non-parametric tests were used. Descriptive analysis was used, including median values, percentages and inter-quartile ranges. Kruskal-Wallis tests were carried out to identify any year group differences with three specific questions: self-rated confidence Part B questions 1) *“I would be comfortable at my stage of medical school education asking a patient about their sexual orientation if I thought it was relevant or necessary”* and 2) *“I would be comfortable at my stage of medical school education asking a patient about their gender identity if I thought it was relevant or necessary”* and Section 5 “*I have received specific training on LGB&T health issues in undergraduate teaching at medical school”.* Ethics was obtained from the Brighton and Sussex Medical School Research Governance and Ethics Committee (ER/BSMS6589/2).

## Results

### Participants

The survey was sent to 776 medical students of which 292 surveys were returned (37.6% uptake rate). 58 of the responses were paper surveys and the rest were completed online. Data analyses were conducted on 252 surveys, of which 232 were fully completed. The median age of participants was 22 years, 63% were female and 80% were heterosexual (Table 1).

### Self-reported confidence in treating LGBT patients

Participants were ‘neutral’ at clarifying unfamiliar sexual or gender terms used by patients (Table 2). Participants felt unconfident deciding which ward (male/female) a transgender patient should be nursed in, and unconfident in knowing where to find information about LGBT-specific health services in their local area. They were however confident taking a social or sexual history from LGBT patients. Confidence ratings significantly increased with year of study with respect to asking a patient about their sexual orientation (H (4)=26.999, *p* = 0.001) with a mean rank of 90.51 for year 1; 102.53 for year 2; 118.84 for year 3; 134.39 for year 4 and 149.54 for year 5). There were no significant year group differences in confidence when asking a patient about their gender identity (H (4)=5.989, *p* = 0.2*)* (Table 2).

### Attitudes toward LGBT patients

Participants strongly agreed that same-sex attraction and a broad diversity of genders was a natural expression in humans. Participants strongly disagreed that it was more challenging to conduct a physical or genitourinary examination with an LGB patient than with a heterosexual patient, but were ‘neutral’ about it being more challenging with a transgender patient than with a cisgender patient (Table 3).

### Awareness of health issues amongst LGBT patients

Participants thought that most listed health issues were more common (to varying degrees) amongst both transgender and LGB patients (Table 4), with a few exceptions, for example, diabetes and childhood diseases. Participants thought that feeling welcome/accepted in health services was equally less common for both LGB and transgender patients. Not attending breast/cervical screening was viewed as more common for transgender patients, but not perceived to be more common for female LGB patients. Overall participants gave higher endorsements with respect to health issues with transgender patients than LGB patients, meaning they believed the health issues to be more common in transgender patients than LGB patients. This applied to 10 of the 16 listed health issues (Table 4).

Participants were very confident in their awareness of commonly used words such as ‘gay’, ‘lesbian’ and ‘bisexual’, however no terms scored 100% confidence. Participants were considerably less confident about surgical terms such as ‘bottom surgery’, ‘phalloplasty’ and ‘neovagina’ (< 50% of participants reported feeling confident). Only 6.9% were confident with the term ‘serosorting’ with respect to HIV status (Table 5).

Participants were asked to consider reasons that might negatively impact on a LGB or transgender person’s attendance at healthcare services. Commonly selected were ‘worries about discrimination’ (99%, *n* = 232), ‘previous negative healthcare experience’ (98%, *n* = 231), ‘worries about feeling unwelcome’ (94%, *n* = 220) and ‘worries of ignorance of the healthcare provider about their specific health needs’ (90%, *n* = 212). Less frequently selected options included ‘fear of healthcare staff’s overemphasis of LGBT identity’ (80%, *n* = 188), and ‘failure of the healthcare system to provide appropriate screening invitations’ (71%, *n* = 166).

### Views on the undergraduate curriculum

Participants were asked if they had received specific training on LGBT health issues in their current undergraduate medical curriculum, the majority (69%: *n* = 161) across all years “disagreed’ or “strongly disagreed”. There was a significant difference (H (4)=43.401, *p* = 0.001) between year groups in how this question was answered. There was a trend towards exposure to LGBT training to increase with year of study with a mean rank of 83 for year 1; 76.07 for year 2; 114.34 for year 3; 132.64 for year 4 and 147.33 for year 5. The majority of students thought the amount of LGBT related teaching was not enough (at 86%: *n* = 199 for LGB and 91%: *n* = 210 for T respectively). Likewise the majority of students (85%: *n* = 198) were interested in more teaching opportunities if they were made available, either distributed across the current curriculum or to a lesser extent confined to specific units such as lectures or seminars. Only 13% (*n* = 30) reported not wanting more teaching on LGBT issues.

Most participants (66%: *n* = 152) reported that social influences were the main source of their current knowledge of LGBT health experiences. The medical school’s core curriculum was endorsed as the second source for learning about LGBT health, with only 17% (*n* = 39) reporting that this had been their main source of information about LGBT health. Participants felt that the most beneficial way to learn about LGBT healthcare was within communication skills training (36%: *n* = 83) or through focus groups or talks directly involving people from LGBT communities (23%: *n* = 53).

## Discussion

This study found that medical student attitudes towards LGBT patients were positive but awareness of health issues and confidence with respect to LGBT healthcare was variable. Self-perceived confidence discussing patient sexual orientation significantly increased over the 5 year course but confidence with respect to discussing patient gender identity did not. The majority of participants reported a clear deficit in the amount of LGBT health training they had received and expressed an interest in receiving more.

### How could the curriculum be enhanced?

Only a small proportion of students reported the curriculum as being the main contributor to their current knowledge of LGBT health issues. This is unsurprising as the majority of students reported a lack of specific LGBT training at medical school. Specific training within a medical curriculum is associated with better knowledge and awareness of LGBT-specific health issues [22, 23] although it would appear that awareness of LGBT issues amongst medical students has improved since previous research in the UK [12].

The stated benefits and the clear preference for more teaching clearly support a curriculum enhancement, which echoes a previous study of healthcare students [24]. The majority wanted teaching spread throughout the curriculum delivered by members of the LGBT community, within an interactive forum. These preferences are supported by Solotke *et al* (2017), who formulated ‘tips’ for including sexual and gender minority health within medical school curricula [25]. They suggest that content should be spread across the curriculum and added to the existing teaching, requiring minimal effort. This is a time efficient solution to an already stretched curricula, and the repeated nature could reinforce learning. Furthermore, incorporating this content into many medical specialties helps to broaden awareness beyond the traditional sexual health setting. This guide also advises to ‘empower allies’, making students feel like they should be involved in LGBT patient care [25]. The interactive learning methods that were favoured in our study would empower students to apply these skills to their clinical practice. This is consistent with a recent systematic review, which argued that face-to-face interactions and clinical exposure to transgender patients were the most effective methods of student learning [26].

Differences in participants’ responses towards LGB and transgender health were observed. Confidence with respect to sexual orientation increased with year of study, but not with respect to gender identity. More students reported a lack of teaching about transgender health compared to LGB teaching which is consistent with the literature [24, 27].

### Strengths and weaknesses

Items relating to LGB and transgender health were separated where possible within this study, contrasting with other UK studies where LGB and transgender health were combined [12]. Few studies have directly assessed medical students’ attitudes and knowledge towards transgender patients and therefore our study provides a more detailed assessment [26]. As far as we are aware our study contains the largest cohort of participants from the UK. In addition to providing direct comparisons with previous research, which used a similar survey [12], we also garnered opinions about a UK medical undergraduate curriculum, an area which has been sparsely researched. It is unlikely that our particular curriculum differs significantly from other UK curriculums in this respect.

In terms of weaknesses, the majority of participants who partially completed the survey stopped at the ‘attitudes toward LGBT patients’ questions. This may have been because of the survey format or the nature of the questioning. The results may be biased due to the opt-in nature of the study, with nearly 20% of participants identifying as gay, lesbian and bisexual. The results may not be generalizable across the UK as participants came from one medical school.

## Conclusions

We identified deficits in medical students’ confidence treating LGBT patients and knowledge of their specific health problems. Despite this, their attitudes towards these patients were positive. Participants reported inadequate training regarding LGBT-specific healthcare, with a strong preference for more. Transgender healthcare appeared to be an especially neglected area. Providing more training for students is an opportunity to break down healthcare barriers that exist between clinicians and LGBT patients. This training should put a specific focus on transgender healthcare, and addressing subpopulation health issues rather than the LGBT community as a whole. Implementing these changes could contribute to the improvement of LGBT patient outcomes, and create a more equitable healthcare environment.

## Data Availability

The anonymised datasets generated during the current study will be available from the University of Sussex Research Data Repository via Figshare. Llewellyn, Carrie (2020): Attitudes, beliefs and knowledge of LGB&T patients’ health needs and barriers to health services_anonymised.sav. University of Sussex. Dataset. 10.25377/sussex.12452669
